# The Irish Football Player Pathway: Examining Stakeholder Coherence Throughout and Across the Player Development System

**DOI:** 10.3389/fspor.2022.834633

**Published:** 2022-02-14

**Authors:** Liam Sweeney, Áine MacNamara, Dan Horan

**Affiliations:** ^1^Faculty of Science and Health, School of Health and Human Performance, Dublin City University, Dublin, Ireland; ^2^High Performance Department, Football Association of Ireland, Dublin, Ireland

**Keywords:** academy football, talent development, talent pathway, stakeholder coherence, talent development principles, Irish football player pathway

## Abstract

Maximizing the efficiency of the player development system is a strategic priority for any professional football club or association. However, the successful development of a young footballer is largely dependent upon the roles and relationships of the different stakeholders invested in the developmental process. This study examined the level of horizontal (i.e., extent to which stakeholders across a pathway stage work with players in an agreed fashion to optimize their experience) and vertical (i.e., extent to which multiple stages of the pathway are coordinated and build chronologically from previous involvement toward long-term needs) stakeholder coherence throughout the Irish football player pathway following a restructuring of development policies and the implementation of a nationwide academy system between 2016 and 2020 under the Football Association of Ireland's (FAI) Player Development Plan. As a second aim, we explored each of the key stakeholders' alignment to academic talent development principles in order to provide practical recommendations for future player and coach development policies. Accordingly, a series of interviews were conducted with 31 key stakeholders currently engaged in the player pathway. These key stakeholders consisted of parents, coaches and members of the FAI as the National Governing Body for football in Ireland. Data were analyzed using Reflexive Thematic Analysis, with findings highlighting a lack of stakeholder coherence across the pathway, both vertically and horizontally. Stakeholders displayed inconsistency in their understanding of the purpose of the player pathway and its long-term strategic aims, as well as demonstrating poor and incohesive relationships with each of the different stakeholders. Moreover, talent development principles between the different stakeholders appeared well-understood overall, although the practical implementation of several of these principles in applied practice did not appear to exist. Results highlight the need for organizational intervention and structural change across the Irish player pathway to maximize long-term player development in the future. Practical implications for the FAI are discussed and recommendations are made to support optimal player development policies moving forward.

## Introduction

Every year, professional football clubs enroll thousands of youth players into their respective talent development systems, otherwise known as academies (Ford et al., 2020; Williams et al., 2020). Within the academy system, well-funded coaches, scouts, sports scientists, and administrators are employed to engage in the process of developing these high potential youth players (Ford et al., 2020). However, the process of talent development is complex. Indeed, while the development and subsequent success of a talented young player is influenced by a variety of innate, psychological and behavioral factors, the successful progression of a young player is also largely dependent upon their specific talent development environment (Mills et al., 2014a,b). Young players will simultaneously engage with various sporting environments (i.e., grassroots clubs, academies, national representative squads) and stakeholders (i.e., parents, coaches, peers) throughout their time in the talent pathway. Given both the complexity and variation in the environments that young athletes inhabit, Henriksen et al. (2010a,b) emphasize the importance of a holistic ecological approach to development, noting that effective talent development environments are characterized by a high degree of cohesion and cooperation with a considerable focus on organizational structure and culture. Martindale et al. (2007) characterized the key principles encompassing effective development environments for young athletes and identified that (1) developing long-term aims and methods, (2) providing wide-ranging coherent messages and support, (3) emphasizing appropriate development, not early success, (4) providing individualized and ongoing development, and (5) providing integrated, holistic and systematic development as characteristics of effective talent development environments. A shared and coherent understanding of these principles between the stakeholders involved in developing young athletes is vital in maximizing athlete progression to elite senior levels (Curran et al., 2021).

In football, talent identification is primarily concerned with selecting pre-pubertal athletes and talent development focuses on their development as they progress throughout puberty into adulthood (Pankhurst and Collins, 2013). Multiple stakeholders play a role in the talent development process, namely the National Governing Body [NGB] (or academy), the club, coaches, and the athlete's parents (Pankhurst and Collins, 2013). Parents' role in the talent development process has been highlighted regularly within research (e.g., Côté, 1999; Gould et al., 2002, 2006; Wolfenden and Holt, 2005). Parents adopt a leadership role during their child's sampling years of development, shifting to a follower/supporter role during the specializing and investment years of development (Côté, 1999). Parents also provide emotional support as their child experiences the psychological stress and challenges of high-level competition (Côté, 1999), which is vital given the non-linear and challenging nature of the “rocky road” to the top (Collins and MacNamara, 2012). Alongside parents, there is a plethora of research attesting to the central role of coaches in supporting talent development in youth sport (i.e., Wolfenden and Holt, 2005; Henriksen et al., 2010b; Abraham and Collins, 2011) and within youth football specifically (i.e., Smith and Cushion, 2006; Cushion et al., 2012; Larsen et al., 2013; O'Connor et al., 2018). Within football, coaches have significant influence and control over player development and the sociocultural dynamics of the learning environment (Cushion et al., 2012). The actions of the coaches impact the behavior, cognition, and affective responses of players, and have a marked influence on young players' overall development (Cushion et al., 2012). Crucially, coaches are often the individuals responsible for identifying talent, although their role in this talent identification process is influenced by their experiences in a specific coaching culture (Lund and Söderström, 2017). The NGB, as the third stakeholder and “system controller,” has responsibility for the policies and systems in the sport, including the coach education system (Pankhurst and Collins, 2013). However, in comparison to the other two stakeholders, the role of the NGB in talent identification and development has attracted relatively less research attention.

The extent to which there is alignment in perceptions across each stakeholder is another crucial element in player development. As Pankhurst and Collins (2013) highlight, the success of a young athlete depends upon each key stakeholder deploying their specific skills, having commonality in their knowledge of athletic development, and an understanding of the talent identification and development process itself, in addition to the existence of quality relationships between each of these key stakeholders. A lack of coherence between stakeholders can impact a young player's success as a result of mixed messages, confused agendas and a lack of clear direction and directives (Pankhurst and Collins, 2013). Indeed, coherence is a characteristic of effective talent development environments (Martindale et al., 2007), represented by inputs that are structured, complementary and framed against long-term agendas (Webb et al., 2016). In essence, a coherent operating system is the strategic center of an efficient development environment, and a cohesive philosophy between stakeholders, characterized by clearly defined core values, expectations, and behavioral standards, represents optimal developmental conditions for young football players (Mills et al., 2014a). Stakeholder coherence can be seen horizontally where athletes experience complimentary coaching and adaptive support based upon changing demands, and coherence can also be seen vertically, where multiple stages of the pathway build chronologically from previous involvement toward long-term aims (Taylor and Collins, 2021).

In an investigation into the talent development environments of elite football academies in Sweden, high-quality environments were characterized by well-established relationships between key stakeholders, whereas in low-quality environments, academy players experienced deficiencies in their support network with a lack of established relationships between stakeholders, particularly between coaches and parents (Ivarsson et al., 2015). In a similar investigation framed within a UK context, Mills et al. (2014a) qualitatively examined the factors perceived by ten expert coaches from ten professional football academies to underpin optimal talent development environments specific to football. The findings highlighted the need for an integrated approach to talent development that establishes a strong link between players, parents and club staff. In essence, optimal player development environments appear to be, at least in part, driven by the existence of coherent stakeholder relationships. In this regard, Pankhurst et al. (2013) note that a lack of stakeholder coherence is a barrier to long-term athlete development, and the chances of success for a young athlete appear to be enhanced if all the stakeholders in the development pathway have similar perceptions of the key elements of talent identification and development, and therefore, have similar behaviors and support systems. Unfortunately, a lack of organizational proximity and communication between youth and professional environments has been observed in many elite football clubs across Europe, hindering youth players' progression into professional football (Relvas et al., 2010). Likewise, several recent investigations into the talent pathways of football academies in the UK have identified a lack of horizontal coherence across the pathway, characterized by low levels of communication between coaches and parents (Harwood et al., 2010; Clarke and Harwood, 2014; Mills et al., 2014b).

Whilst the knowledge, skills, and abilities of each stakeholder are unique and specific to the athlete, there is a need for consistency and clarity in messages and support between the stakeholders if the potential of a player is to be realized (Martindale et al., 2005; Pankhurst et al., 2013). In most talent identification and development systems, stakeholder understanding of the fundamentals of the key constructs of any process is presumed to exist, although research thus far does not appear to support this presumption (Pankhurst et al., 2013). However, there is a lack of empirical evidence about how coherence can be realized in regard to the methods, structures, and opportunities that young athletes are afforded as they progress within a complex talent pathway. As a result, there have been calls for further research to provide clarity in this area (cf. Pankhurst and Collins, 2013; Pankhurst et al., 2013; Webb et al., 2016). Indeed, there is a growing research base that has sought to examine talent development environments in a youth football context (i.e., Larsen et al., 2013; Aalberg and Sæther, 2016; Flatgård et al., 2020; Larsen et al., 2020). However, despite providing valuable additions to the talent development literature, these investigations have predominantly focused on a single football club or age group in a non-Irish cultural context. Whilst similarities in talent development environments can exist at their core, development environments are highly individualized and culturally specific (Henriksen et al., 2010a; Mills et al., 2014b). Examining the cultural context of talent pathways is especially important given the over-arching and systematic influence culture has on the development of talent (Martindale et al., 2007).

The Football Association of Ireland (FAI) is the NGB for football in Ireland. Between 2014 and 2019, the FAI introduced the under 17, 15, and 13 (changed to under 14 in 2020) underage National Leagues (NL), respectively, to enhance the development of players in Ireland. The introduction of the underage NL required senior NL clubs (also referred to as League of Ireland clubs) to create affiliated academy squads from the under 13 to under 19 age cohorts, similar to the academy system seen in professional clubs elsewhere throughout Europe. Before the introduction of the underage NL, no academy system in Ireland existed and schoolboy football clubs (also referred to as grassroots football clubs) were responsible for the development and transfer of players to professional clubs abroad, particularly to clubs in the UK. Schoolboy football clubs are governed by the Schoolboy Football Association of Ireland (SFAI), which is an affiliate of the FAI. The SFAI has 30 separate football leagues throughout Ireland, with each schoolboy football league also governed independently by their individual league council. This makes the dynamic of the Irish football landscape particularly complex and cumbersome, with each schoolboy football league operating independently from one another under the jurisdiction of the SFAI, and the SFAI operating independently from the FAI. Prior to the introduction of the NL, large schoolboy clubs in the Dublin District Schoolboy League (DDSL^1^) dominated schoolboy competitions and, due to the number of players from this select number of grassroots clubs signing for professional clubs in the UK, were considered by many to be best clubs for high potential players from around Ireland to play for. This led to players from different parts of Ireland traveling large distances to play in the DDSL. The NL were introduced by the FAI to provide a consistently higher quality of youth coaching and competition nationwide so that players in every county had the opportunity to avail of coaching and playing environments that are better suited to their needs. This decision was a radical intervention in player development in Ireland and caused significant unrest among the SFAI and many of its member clubs.

This study aimed to explore the extent of horizontal and vertical coherence across the Irish football academy pathway and to explore the key stakeholder perceptions of, and alignment to, talent development principles. Given the lack of previous research investigating how the introduction of the new Irish football academy pathway impacts each stakeholder, the nature of this research was considered timely. As Mills et al. (2014a) note, “[our] knowledge of athletic development environments is far from complete, particularly where elite youth soccer is concerned” (p. 138). To bridge this gap, qualitative interviews were conducted with the key stakeholders across the Irish football pathway: parents, coaches (from both schoolboy clubs and NL clubs), and the FAI as the NGB. This was the first research of its kind to critically and comprehensively analyse the extent of stakeholder coherence across the Irish football academy pathway, and consequently, this research was considered necessary to optimize future player development and coach development policies within the FAI.

## Methodology

### Research Philosophy

Given the purpose of this study and our aim to generate practically meaningful knowledge, this study was grounded by a pragmatic research philosophy. A pragmatic research philosophy is one that employs methods with the aim of answering questions and providing practical solutions, rather than being driven by a distinct epistemological approach (Giacobbi et al., 2005). Pragmatic approaches also suggest the prioritization of questions and methods that are practically meaningful rather than seeking generalisable truths or subjective constructions (Taylor and Collins, 2021). Pragmatism maintains that researchers are not passive observers, and as such, it was important to note that all authors have experience working with talent pathways and in the context under investigation in particular. Our positioning as practitioners familiar with the context facilitated novel and innovative insights (Bryant, 2009) and formed the platform for a detailed enquiry, allowing us to combine our applied experience with relevant literature, considering ourselves as co-constructors of knowledge, attempting to generate meaningful information. Reflecting our pragmatic approach and the aim of this research, a qualitative methodological approach in the form of one-to-one interviews was adopted to allow for an in-depth investigation of the perceptions and experiences of the pathway from the perspectives of each individual stakeholder. Underpinned by this approach, we embraced the experiences, realities, and reflections of each stakeholder engaged in the Irish football player pathway.

### Participants

Thirty-one participants were purposefully recruited based on their current involvement in the Irish football pathway. Participants were subdivided into separate samples of parents [P] (*n* = 9) aged between 44 and 53 years (M = 49.4 years; SD = 3.0 years), Schoolboy football club coaches [SC] (*n* = 8) aged between 33 and 58 years (M = 43.4 years; SD = 7.3 years), NL academy coaches [NLC] (*n* = 10) aged between 23 and 53 years (M = 35.8 years, SD = 9.3 years), and coaching/coordinating personnel from the FAI underage national coaching system [FAIC] (*n* = 4) aged between 30 and 65 years (M = 42.3 years; SD = 16.5). All participants were recruited from an evenly distributed geographical sample to provide a nationally representative spread of participants across the country. Moreover, such a spread of coaches was selected to provide a representation of all levels of Irish underage football, from schoolboy level through to the national level, with each coach deriving from a different club. All coaches were coaching at the under 14 to under 17 age groups at the time of data collection, and all parents had a child currently signed to a NL academy in the pathway at the under 14 to under 17 age groups. Given the introduction of the new underage NL and academy infrastructure in Irish football under the (Football Association of Ireland Player Development Plan, 2015), each participant's engagement in the pathway under the current system ranged from 1 to 5 years at the time of data collection.

Our research focussed on the player development pathway for male players only, as at the time of data collection, the under 14 and 15 NL existed for male populations only. Following ethical approval by the first author's institutional Research Ethics Committee, all participants were invited to participate through personal contact *via* gatekeepers within the League of Ireland and at the FAI. After agreeing to participate in the study, all participants were contacted by email and, where necessary, by telephone by the first author, informed of the purpose of the investigation and assured of confidentiality. All participants were provided with a participant information document before providing informed consent.

### Data Collection

To maintain relevance to the research questions, a semi-structured interview guide with open-ended questions and relevant follow-up prompts was reflexively adhered to. The interview guide was underpinned by relevant literature and the authors' knowledge and experiences of Irish football and talent development environments. Questions revolved around various experiences and perceptions of the Irish football pathway (i.e., What is your understanding of the FAI player pathway? What are the objectives of the underage National Leagues? What are the coaches from the National League clubs looking for in players during talent identification?), with probes and prompts used to clarify and expand on specific points (i.e., Are these the right things in your opinion? How does that influence what you do in your role?). The interview guide was tested and refined through pilot work with three Irish football coaches aged between 38 and 55 years (M = 44.3 years; SD = 9.3), all of which were qualified at a minimum of the UEFA A coaching License, with between 4 and 25 years of coaching experience (M = 9.3 years; SD = 10.5 years). The interview guide is available on request from the first author.

Interviews were conducted between each participant by the first author. Interviews were conducted electronically *via* video interviews using the Zoom video software (Zoom Video Communications, San Jose, California, USA) to ensure adherence to the national government's COVID-19 pandemic safety regulations at the time of data collection. In instances where face-to-face interviews are unfeasible, Zoom electronic video interviews are recommended as an alternative method to gather rich data, whilst facilitating a positive participant experience (Gray et al., 2020). Interviews were conducted with the first author and each participant in a quiet location, and a pre-briefing allowed participants to reflect upon the themes to be discussed and provided an opportunity for further questions. Excluding an initial briefing and warm-up questions, interviews lasted between 43 and 81 min (M = 60 min; SD = 10 min) and were video and audio recorded for subsequent transcription and analysis. Manual transcription was conducted by the first author, with transcription documents then re-checked against audio recordings to confirm transcription accuracy.

### Data Analysis

Reflexive thematic analysis (RTA) (Braun and Clarke, 2019, 2021) was conducted to analyse the content of the interviews. RTA “is about the researchers reflective and thoughtful engagement with their data and their reflective and thoughtful engagement with the analytic process” (Braun and Clarke, 2019, p. 594). Cohering to the interpretive style of RTA, analysis was predominantly conducted by the first author, and consequently, the results of the analysis reflect the first author's interpretation of data. Given the nature of RTA (cf. Braun and Clarke, 2019, 2021), interviews were conducted with flexibility and fluidity to resemble the flow of a real-world conversation, with considerable scope for the researcher to be spontaneously responsive to the participants' unfolding accounts. Such an approach allowed the author to gain an in-depth exploration of each participant's story, rather than a uniformly structured account. This flexible and fluid approach to interviewing was considered appropriate for extracting each participant's perceptions, beliefs and experiences of the Irish football player pathway. Using an inductive “bottom-up” approach, data was open-coded with a focus on deriving semantic and latent codes and themes strongly linked to the data itself. However, deductive analysis was also employed to ensure that open coding produced themes relevant to the research question. An experiential orientation to data interpretation was adopted to emphasize meaning and experience expressed by participants, and epistemological theoretical considerations for analysis were essentialist, meaning language was interpreted as a reflection of participant meaning and experience.

Data was analyzed using Braun and Clarke's (2019, 2021) six-phased approach to RTA. Data was manually and orthographically transcribed to Microsoft Word (Windows Microsoft, Washington, USA) to facilitate deep immersion in the data, each transcript was then re-read several times, and familiarization notes were taken to ensure familiarity and understanding of the data before systematic data coding began. Once data was coded, initial themes were generated from the codes. Potential themes were then developed and reviewed and structured into a framework of higher-order themes, followed by the refining, defining and naming of themes based upon the content of the codes within each theme. The sixth and final phase consisted of writing the report, although given the reflexive nature of RTA (Braun and Clarke, 2019, 2021), report writing was recursive and woven into the entire process of the analysis. The qualitative analysis software (QSR NVIVO-12) was used to assist in the structuring, organizing and analysis of raw data into their thematic hierarchies.

### Trustworthiness

The first author was experienced in the Irish football environment at NL level which brought familiarity and awareness with participants on the topics being discussed throughout each interview. These factors helped build trust and rapport with participants which supported the breadth and depth of information provided. Throughout data analysis, the first author kept a reflexive journal to record their reflections and insights throughout data collection, and also to use the practice of writing as a tool for deepening reflexivity (Braun and Clarke, 2021). Given the interpretive and reflexive nature of RTA, analysis was predominantly conducted by the first author, with the second author auditing the reflexive analytical process by sense-checking analysis and exploring other alternative interpretations of data. This process encouraged reflexivity by challenging the first author's construction of knowledge in a way that was collaborative and reflexive, aiming to achieve richer interpretations of meaning, rather than to achieve consensus of meaning (Braun and Clarke, 2019; Byrne, 2021).

## Results and Discussion

This study aimed to examine the extent of horizontal and vertical stakeholder coherence across the Irish football academy pathway and to explore the key stakeholder perceptions of, and alignment to, talent development principles. The RTA produced two themes (stakeholder coherence and alignment to talent development principles), seven higher-order themes, and 15 lower-order themes which are displayed in Table 1. These higher-order themes and lower-order themes are subsequently presented in detail with exemplar quotations below to illustrate the analysis.

**Table 1:** The themes, higher order themes, and lower order themes produced from the reflexive thematic analysis.

**Theme**	**Higher order themes**	**Lower order theme**	**Raw data theme**	**Mentioned by participants (%)**			
Stakeholder coherence	Vertical coherence	Lack of shared understanding regarding the purpose of the	Generate finances from selling players to clubs abroad	40% NLC	75% SC	56% PAR	25% FAIC
		player pathway	Provide a pathway to first team football	80% NLC	25% SC	33% PAR	25% FAIC
			Produce players for Irish international teams	60% NLC	20% SC	33% PAR	75% FAIC
			Provide an environment for the best players to play with and against the best players	50% NLC	38% SC	44% PAR	25% FAIC
			Increase the standard of domestic football	50% NLC	13% SC	11% PAR	25% FAIC
		Disjointed relationships between National League and schoolboy clubs	The poor relationship between National League clubs and schoolboy clubs is hindering player development	100% NLC	100% SC	89% PAR	75% FAIC
			Schoolboy clubs are reluctant and uncooperative in transferring players to the National League clubs	90% NLC	50% SC	78% PAR	75% FAIC
			Schoolboy clubs dislike the loss of power caused by the introduction of the National Leagues	70% NLC	33% SC	56% PAR	75% FAIC
			National League clubs inappropriately approach players without cooperating with schoolboy clubs	20% NLC	25% SC	33% PAR	25% FAIC
			The misalignment in youth football seasons is hindering player development	0% NLC	25% SC	22% PAR	50% FAIC
		Poor relationship between the FAI and the SFAI	The FAI and SFAI do not cooperate or communicate	30% NLC	38% SC	44% PAR	50% FAIC
		Conflicting opinions regarding the quality of coaching throughout the pathway	The standard of coaching in the National Leagues is optimal for player development	100% NLC	25% SC	100% PAR	0% FAIC
			The style of play being coached in the National Leagues is poor	10% NLC	63% SC	0% PAR	25% FAIC
			Higher quality coaching needs to begin at earlier ages	60% NLC	13% SC	22% PAR	50% FAIC
			The quality of coaching at the schoolboy level is generally poor	90% NLC	63% SC	56% PAR	25% FAIC
			The quality of coaching at the schoolboy level and in the National Leagues is the same	0% NLC	38% SC	0% PAR	0% FAIC
	Horizontal coherence	Poor relationships between coaches and parents within	Parents of academy players can be overbearing	80% NLC	0% SC	33% PAR	50% FAIC
		National League academies	Lack of communication from coaches to parents	0% NLC	0% SC	56% PAR	0% FAIC
			Lack of parental education	10% NLC	25% SC	22% PAR	25% FAIC
			Open and regular lines of communication exist between National League coaches and parents	50% NLC	0% SC	11% PAR	0% FAIC
		Concerns over the youth to senior transition	There is a divide between academy teams and the senior departments at National League clubs	50% NLC	13% SC	22% PAR	50% FAIC
Alignment to talent development	Early engagement	Football player engagement	Academy players have given up other sports to focus on football	70% NLC	0% SC	56% PAR	0% FAIC
principles			When players enter the academy pathway the majority of their sporting activity should be devoted to football	60% NLC	25% SC	44% PAR	75% FAIC
			Football free play is essential for development	40% NLC	13% SC	11% PAR	0% FAIC
		Opinions about engagement in other sports	There are biopsychosocial benefits of playing other sports	90% NLC	63% SC	89% PAR	100% FAIC
			Academy players should be encouraged to recreationally participate in other sports on non-football days	50% NLC	0% SC	33% PAR	75% FAIC
			Sampling other sports is important during the childhood years of development	70% NLC	75% SC	89% PAR	100% FAIC
		Irish culture	The GAA is an important part of Irish culture	70% NLC	63% SC	44% PAR	25% FAIC
			Irish culture is multiple sport participation	50% NLC	38% SC	11% PAR	0% FAIC
	Systematic barriers to	Lack of appropriate resources in the National Leagues	Lack of facilities	70% NLC	75% SC	22% PAR	100% FAIC
	player		Lack of financial investment in Irish football	40% NLC	38% SC	22% PAR	25% FAIC
	development		Lack of a full-time football industry	90% NLC	13% SC	0% PAR	25% FAIC
	Biological maturation	Early maturation selection biases	Player selection is based upon physical attributes	30% NLC	63% SC	89% PAR	0% FAIC
			Later developing players are overlooked in selection	20% NLC	50% SC	33% PAR	0% FAIC
		Lack of developmental opportunities for later maturing	National League age gaps hinder later maturing players	30% NLC	38% SC	22% PAR	50% FAIC
		players	Players should be matched by physical size rather than age	20% NLC	13% SC	11% PAR	0% FAIC
	Lack of	Uncompetitive matches	Large score-lines and uncompetitive matches	50% NLC	50% SC	56% PAR	25% FAIC
	appropriate	Lack of contact hours	Lack of training hours	70% NLC	13% SC	33% PAR	50% FAIC
	challenge		Lack of matches	20% NLC	25% SC	0% PAR	0% FAIC
	An emphasis on short-term success	A focus on short term results	A focus on winning matches	70% NLC	38% SC	56% PAR	50% FAIC

*FAIC, Football association of Ireland coaches; NLC, National League academy coaches; PAR, Parents; SC, Schoolboy football club coaches*.

### Stakeholder Coherence

#### Horizontal Coherence

A lack of horizontal coherence was apparent at the NL level, as evidenced in the relationships between coaches and parents. Eight NL coaches cited experiences of interference from parents, specifically parents applying excessive amounts of pressure on their children and parents providing contradictory and inappropriate coaching advice. The majority of coaches cited overbearing parents as one of the biggest barriers faced at the internal club level, and explained how these parents hinder their ability to perform their role as coaches effectively:

“*The first year was tough. I had parents who were quite opinionated, who were vocal on the side-line, who would completely contradict the information that the players were getting from us in their eight hours in training. We spend eight hours a week or five hours with the kids and we would be telling them this and then the parents would completely change that and then the player becomes quite conflicted in what to do”* [NLC6].

“*I've seen it with young lads, and nobody is perfect, and we all have bad days and sessions and things like that, but to see a kid get into a car with a parent and the parent tears strips off of the child because they missed a pass or missed a goal or something like that, that's where you as a coach have to be going to the parent and saying ‘that’s not your job, its mine. I'll talk to him' (…) some kids are afraid for their life!”* [NLC3].

Côté (1999) highlighted the important role of parents during athlete development through the provision of emotional support as their child experiences the psychological stress and challenges of high-level competition. The results here indicate that a proportion of parents are behaving in ways that can negatively influence long-term athletic development (i.e., providing inappropriate coaching advice, putting pressure on their child). Similar behaviors have also been observed in the parents of English academy players by Mills et al. (2012) and highlight the need for further and more comprehensive parental education; another theme that emerged from this research. Six participants commented on the need for more parental education to help parents understand their role as “football parents,” but also to help parents understand the roles of the coaches and other key stakeholders. For instance, as the child enters the academy at the specialization stage of development, the responsibility for the child's sporting development transfers from the parent to the coach, and parents must accept this transference of responsibility and adopt a role of support for their child (Côté, 1999; Clarke and Harwood, 2014). This need for parental education was exemplified by the reflections of P1:

“*I also think parent education [is needed], because people think that shouting on the side-lines at their son or at the referee…let me tell you, I was guilty of it and I am horrified by it, the kids feed off of that. If you're going to bring kids through football and it's about the journey, parents are inextricably linked to the child. The kid gets brought every day, and the chances are that it's the father in my experience, but the fathers are the worst, they are the absolute pits of it, and there has to be an educational piece for them to understand because they're the ones on the side-lines dictating.”*

The need for parental education was reciprocated among NL coaches:

“*I think more can be done with parents to help them understand it. I find that there is a lot of, well maybe not a lot, but there are parents that want the success now. If their children aren't being looked at for Irish assessments, or if their children aren't starting every game, or if the child is coming off after 30 minutes in every game, the parents seem to want answers*” [NLC4].

Indeed, the need for parental education and regular coach-parent communication within football academies has been highlighted elsewhere (Harwood et al., 2010; Clarke and Harwood, 2014; Newport et al., 2020). Although NL coaches in this study claimed that they do have open and regular lines of communication with parents, the majority of parents were critical of coach communication and believed the level of communication from coaches to be insufficient. This has also been observed in the parents of academy players in the UK, where parents felt that they received inadequate communication and were underappreciated in their role as parents by coaches (Harwood et al., 2010; Clarke and Harwood, 2014). Mirroring these findings, parents in this study expressed a desire for more regular and comprehensive communication from coaches, as P8 explained:

“*There is a certain vacuum around communication with the club…they're not great, their engagement isn't great with the parents if I'm being honest. They give you the bare minimum and they expect a lot with the kids to be there three days a week for two hours and they work them really hard. You're travelling every weekend. They could manage the parent piece better.”*

Ten participants also raised concerns over the youth to senior transition in the NL. Specifically, participants expressed how they believed there to be a divide between the senior hierarchy and underage teams within NL clubs, with clubs viewing their underage teams as an inconvenience rather than in a positive developmental light, as NLC1 explained:

“*What I would say is that certain [National League] clubs do not take it serious and probably only have underage teams to make sure they get their licence, and that would be a massive criticism of some clubs; that you know when you're going to play them that they're not really bothered about how their under 14s or 15s get on, it's just something they have to do to tick a box to make sure they get a licence. And there would definitely be some clubs that if you offered them or said to them ‘you don’t need a 14s, 15s, or 17s and you'll still get your license', I'd say they'd get rid of them in a heartbeat.”*

A lack of coherence between youth academies and senior departments within professional football clubs is not uncommon and is purported to hinder players' youth to senior transition (Relvas et al., 2010). Participants also raised concerns over how the current alignment of age structures within the NL prevented an appropriately staged increase in challenge for young players and was too big of a jump for many players. In response, participants expressed the need for an intervention to bridge the gap between youth and senior football at the exit phase of the pathway:

“*The only other issue I would have is at the top end of the pathway at the under 19s with keeping the players a wee bit longer. Most clubs do not have a reserve team or a league they can put them into so there is no point in us having everything brilliant at the bottom of the pathway if it gets to the end and it's bottlenecked” [NLC10]*.

“*For me, rather than letting players go at 19 years of age and bringing them back and signing them at 21, 22, 23 years of age, why can't we have that under 21s and… should we be bridging that gap there? That's my own thoughts on it”* [NLC8].

#### Vertical Coherence

All participants reported how a disjointed and fractious relationship existed between NL clubs and schoolboy football clubs. Specifically, twenty-nine participants explained how this fractious relationship was the most fragmented element of the player pathway and was subsequently hindering long-term player development. Typifying this, NLC10 commented:

“*There is no relationship between the schoolboy's football and the league of Ireland clubs up here, it's non-existent. It makes our job more complicated every year. It's more work for me and my coaches, and more work for the schoolboys as well, but who loses out? It's the players.”*

The disjointed and incoherent relationship between schoolboy clubs and NL clubs was considered to be hindering player development by all schoolboy coaches, as exemplified by SC8, who expressed the need for urgent intervention:

“*I could go on and on about it, but it needs someone to go in and grab it by the scruff of the neck, and it would need to be a high profiled guy that has a bit of common sense and has a love of the grassroots and has an interest in the league of Ireland and has an interest in seeing the elite players progress and to have a proper pathway. At the moment, it is a bit disjointed.”*

The majority of parents and FAI coaches reiterated that this divide in the pathway was negatively impacting long-term player development. Mills et al. (2014a) described a coherent operating system as the center of an optimal football player development environment, emphasizing the need to develop positive working relationships with both internal *and* external stakeholders. Our findings indicate that NL academies have not established strong cohesive relationships with the schoolboy clubs.

This disjointed pathway was epitomized by the reluctance of schoolboy clubs to allow their players to transfer to NL academies, despite this being the natural vertical progression. Nine NL coaches expressed frustrations over how schoolboy clubs were being intentionally uncooperative in the transfer of players and were actively discouraging players from joining NL academies. Typifying this, NLC3 explained:

“*I won't mention the clubs, but even we have a lot of trouble with a certain club in [Names Location]. They discourage their players from going and playing National League! Because all they want to do is win a youth cup or a national cup and they want to have it on their role of honour that they won that. But in my mind, they're holding back young lads! (…) I just think some clubs, the way they go on and discourage it [the National League], it's not good for young lads, it's not good for the kids.”*

The reluctance of schoolboy football clubs to allow players to progress into NL academies was also cited as an issue for parents:

“*I think what everybody knows, and from my perspective as a parent, my experience of moving from the schoolboys to the league of Ireland was not nice. There was an absolute clash between the junior [schoolboy] club and the league of Ireland club. They were at loggerheads and couldn't agree”* [P1].

“There's resistance, and anecdotally I can tell you that there is this idea that there are people in [schoolboy] clubs who are being very awkward. For example, a certain schoolboy club refused to transfer players by the June 1st deadline just to be awkward. The lads were then effectively on gardening leave and they couldn't play for their league of Ireland club because the schoolboy club didn't sign the forms because they weren't legally obliged to do so. That kind of sh^*^thousery is kind of rife” [P9].

Contrastingly, eight participants, including two NL coaches, explained how NL clubs often contact and attempt to sign players from schoolboy clubs without communicating or seeking approval from the schoolboy clubs first. Similar accusations have historically been made between schoolboy clubs before the NL began. However, this approach by NL clubs was perceived to heighten the conflict between the two entities, as illustrated by SC1:

“*I've had league of Ireland clubs contact parents directly behind my back while the players have been signed to my club. I've had RDO's [Regional Development Officer's] go directly to players (…) I know there have been other trials in other counties where [National League] clubs have not been honest with me, and I still wished those players the best of luck when they left because there's no point in falling out with them because life's too short, but I didn't help that club next time they came asking.”*

Under the current pathway, many schoolboy football league seasons, such as the DDSL, run from September to May, whereas the NL seasons run from March to November, with both seasons having conflicting registration periods. Epitomising the lack of vertical coherence, schoolboy and NL seasons were previously aligned (March–November), but in 2019 several schoolboy leagues reverted back to the September to May season. This season misalignment was suggested as a barrier to player development by six participants and meant that schoolboy clubs were subject to losing numerous players once the NL seasons begin, resulting in uncertainty over playing squads and likely heightening the reluctance of schoolboy clubs to let players transfer to NL club's mid-season. Such frustrations can be demonstrated by SC4:

“*One of the big things is that we need to get alignment between schoolboy football and National League football seasons because at the moment it is sort of farcical. I look at my age group at under 14, we will start off the season great, we will have 14 teams in the league, we get to December or January and the likelihood is we will lose several teams and there will be a big gap of players because other league of Ireland clubs will take players from the DDSL.”*

The under 14 Kennedy Cup, an interleague competition for the 30 schoolboy leagues in Ireland, has traditionally been seen as a shop window for the best players from the 30 schoolboy leagues in Ireland to attract the interest of professional clubs in the UK. The introduction of the NL at under 13/14 meant that many of the highest potential players in the country at that age were playing for NL clubs and were no longer available to play for their schoolboy leagues in the Kennedy Cup. Participants expressed a belief that this loss of control and the transfer of power to the NL clubs has caused feelings of envy and jealousy amongst many schoolboy clubs who traditionally would have held control over player development in Ireland. This is exemplified by the reflections of NLC6:

“*Historically, the schoolboy clubs in Dublin would have produced the players and they still feel aggrieved that they got it taken off them overnight. They feel that they can produce players and they do have a history of producing players. (…) So, I think that's it, it's a loss of control and a loss of power.”*

This divide between schoolboy and academy football was perceived to be caused by the poor historical relationship between the FAI and SFAI. This poor relationship was characterized by a lack of cooperation and communication, with an unwillingness to establish positive working relationships:

“*I think the old sort of analogy of you've got so many kings, and no one wants to lose control of their own palace or their own castle or whatever. I'm assuming, well I'm sure that there's a lot of historical issues there. I think there may potentially be some issues between the SFAI and the FAI which is obviously not allowing them to sit down and come together to create what the best solution is for player development in this country. So, I think you've got a massive issue there”* [FAIC3].

A lack of coherence was also apparent in the disparity of participants' understanding of the purpose of the NL. Parents explained how they felt that NL clubs were focusing on player development, but this focus was based on producing as many players as possible to be sold to foreign clubs to generate finances, rather than for the wider benefit of Irish football, as P3 explained:

“*The progressive Irish clubs will be looking for players with skill, but I fear it might be just to make a killing on the player. I think they're thinking that if a player comes good, they could take in loads of them and even those who aren't good and they can coach them, and then get them flogged off to an English club and make money. I think a lot of it is down to money.”*

The majority of schoolboy coaches agreed, expressing a belief that the reason for the implementation of the NL was economically driven, as SC5 explains:

“*My opinion on it [the National Leagues] a couple of years ago hasn't changed from what it is now; I think it was a way for the FAI to get money into the league of Ireland clubs because if you look at the money certain [schoolboy] clubs were getting for getting players over to England, like one of the north side clubs would have been doing very well with that and would have been pocketing a fair few bob. In my opinion, at the beginning, I felt it was about getting money into the league of Ireland clubs without having to do it themselves, and I still would be of the same opinion.”*

With young players transferring from schoolboy clubs to NL clubs at earlier ages, this reduces the proportion of the transfer fee schoolboy clubs receive if a player transfers to a professional club abroad. This may heighten tensions between both entities over player transfers. Contrastingly, FAI coaches took an opposing view and the majority indicated that the NL were introduced to create an environment that would assist in developing players capable of representing Irish international teams:

“*To give them a roadmap for their development so they can continue to improve and ultimately allow them to not only achieve whatever their potential may be but hopefully to become a professional player and also to assist and to create better players for our underage and senior international teams”* [FAIC3].

NL coaches provided a variety of responses that differed from that of schoolboy coaches and parents. NL coaches indicated that the two primary goals of their club were to produce players for first-team football; “*For me, as head of academy at my club, my sole interest would be to get players into the first team at our football club”* [NLC10], and to produce players for Irish international teams; “*I suppose it's getting better players to represent Ireland at international level”* [NLC4]. Moreover, NL coaches cited additional reasons for the implementation of the NL, including to create an environment for the best players to play with and against each other consistently, and to increase the standard of domestic football.

In respect to coaching standards in the NL, all parents reported that the coaching quality was overwhelmingly positive and were complimentary about how their children were developing as footballers since joining their respective academies. Parents were very happy with the coaching quality, citing it as, for example, “*second to none”* [P8], “*as good as it gets”* [P7] and “*top-level, really, really good*” [P3]. In particular, parents were encouraged by the high-quality and individualized approach to coaching. Typifying this, P9 commented:

“*I have to say that excellent is the word that I would use for this coach and his backroom team. It's innovative, they don't get bored, he has them doing the serious stuff and there's a novelty factor. Organisation and communication, it's all just planned. I don't think he does anything else! He overdoes his job, in a good way.”*

In line with this, all NL coaches claimed that their coaching standards were high and were tailored toward player development. Moreover, NL coaches believed that their coaching was at a higher standard than was being provided in schoolboy football clubs. This may be a reflection of the coach education policies implemented by the FAI. To coach at the underage NL level, coaches must be qualified at UEFA B (under 14) and UEFA A (under 15–17) standards. Although NL coaches commented on how the standard of coaching at certain schoolboy clubs can be good, their impression was that the coaching quality at the schoolboy level is generally poor, mainly due to the reliance on parents/volunteer coaches; “*I just don't think the standards are there, I think that's the big one in that. Grassroots are fundamentally volunteers and you're relying on some volunteers taking things more seriously than others”* [NLC1]. This reliance on amateur coaches at the schoolboy level was perceived to be particularly detrimental to player development from a technical standpoint:

“*I think it goes back to the coaching at the grassroots clubs for me. The big issue that I've had since managing at [National League Club] is the players' technical ability. They may be the better players at their grassroots clubs, and they've got good strengths and attributes, whether that be defending one to one or whatever it is, but I just find they're not technically comfortable” [NLC5]*.

In response to the perceived poor coaching quality at the schoolboy level and the subsequent lack of technically skilled players progressing through the pathway, six NL coaches and two FAI Coaches cited the need for higher quality coaching to begin at earlier ages. Coaches explained how a lack of technically skilled players being developed in Ireland must be solved through earlier intervention by qualified coaches: “*I do believe that there needs to be more work done at a younger age, especially on the technical aspects. (…) It doesn't need to be as serious, but we should be focusing on it from 8, 9, 10 [years of age] in a fun technical way and really improving the technical aspects before they come into the National League”* [NLC9]. Supporting this, NLC10 stated; “*It just makes sense, the younger you can get them and work with them, then the more positive impact you can have on their career and the better player they will be by the time they get to National League football.”*

Contrastingly, five schoolboy coaches were critical of certain coaching aspects in the NL, particularly regarding the styles of play. Schoolboy coaches explained how young players were being “overcoached” and the pressure to fit within tactical systems and set patterns of play was removing the freedom of expression in young players:

“*it's a bit sterile and it takes a bit of the freedom away from the child and has them playing a certain way and the way that they have to play. Whereas, in grassroots football, there is a system, but there is a freedom there to go and express themselves. Its nearly overcoaching on systems and play”* [SC4]

However, five schoolboy coaches did also mention positive aspects of NL coaching, citing it as, for example, “more structured and more detailed” with “more qualified coaches.”

### Alignment to Talent Development Principles

#### Early Engagement

Seven NL coaches stated that the vast majority of players within their club have given up other sports to focus on football. Coaches felt players were deciding to invest in football due to a love for football and to give themselves the best opportunity to maximize their development:

‘‘*I think we have twenty players, and off of the top of my head, three of them play GAA (Gaelic Games) at the same time, other than that, that's it, so for seventeen of my players, their only sport is football. I think if we were at schoolboy, it wouldn't be. I think it's because its National League, they think ‘this is a really good chance for me, and I love the environment and I want to give this everything”’* [NLC6].

Coaches also explained how numerous academy players have given up other sports and now use that spare time to undertake additional football training outside of the academy on non-training days:

“*I know there are loads of players doing extra training and even getting one-to-one coaching on the days that we are not training. So, there are some that are putting just as much time into football as they are putting into school”* [NLC4].

This viewpoint was reiterated by several parents of NL academy players. Parents explained how their children enjoyed playing football and had ambitions to become professional players and, with this in mind, made the decision of their own accord to stop playing other sports:

“*That's his choice [to give up other sports]. I think he's made that choice because he thinks he's a good footballer and he wants to see how far he can go, and he knows he needs to work*” [P5].

Unlike the academy system in countries such as England where players can enter the pathway at age six (Elite Player Performance Plan, 2011), the Irish academy system begins at 13. Participants in this study suggested that when academy players enter the pathway at 13, many decide to focus solely on football. This aligns with Cote's Developmental Model of Sport Participation, whereby at the end of primary school (around age 13), children have the opportunity to specialize in their chosen sport or continue in sport at a more recreational level (Côté and Vierimaa, 2014). However, all parents did reiterate that participation in other sports in their child's school environment was still compulsory, so no players had completely delaminated participation in other sports.

Six participants commented on the important role of football-specific free play in player development. Participants commented on how the lack of elite-level Irish players being produced over recent decades may be correlated to the lack of engagement in football free play by recent generations:

“*Probably, what our players need to do more of is be out in the garden or out with their friends kicking a ball around. That's probably why we as a nation were a lot more competitive 20 years ago; because players were getting so many hours in, not just with teams and coaches, but they were getting them in their back garden or school or whatever”* [NLC5].

Such suggestions mirror the recommendations of Zibung and Conzelmann (2013) and Sieghartsleitner et al. (2018), who proposed that the pathway should provide large amounts of football-specific activity but delivered in broad and diverse developmental formats. Supporting this, NLC2 reflected:

“*There's kids who come out of Brazil who don't get coached and they just play [football] constantly for hours and they just figure things out themselves, and all they play is football all day, all night. They're volleying the ball, they're doing beach football, everything is done with the football. What's the biggest single population that produces footballers in this world? It's the favelas in Brazil, but nobody [coaches] touches them until they're 14, but they play every day and they only play one sport every day.”*

However, the vast majority of participants commented on how the football pathway and decisions surrounding player development policies needed to account for the uniqueness of the Irish cultural context in which the pathway exists. Despite the perceived benefits of early engagement in football, participants cited how the GAA is a unique, but dominant part of Irish culture that goes beyond sport and heavily influences both sporting and societal contexts; “*Gaelic is so predominant, it's so patriotic, it's more than just which sport do you play. (…) Gaelic is just intertwined with social life”* [P4]. Moreover, participants commented on how implementing a player development system similar to those adopted in other successful European football nations would be very difficult, as culturally “*football in Ireland is competing with the GAA”* [P1]. From a player development perspective, such cultural ties with the GAA were seen as a barrier long-term:

“*I have two lads [in my team] who are from here but play down with me in [Names Location] and their school is just hurling. So, their chance of getting football in school is nil. If you took a football onto one of their hurling pitches, you'd get expelled I'd say, it's that serious. So, this is where the problem comes in”* [NLC3].

“*We are unique in terms of some countries because in some countries, all they have is football, whereas we've got Gaelic and Hurling. There's a lot of young people living in country villages and stuff like that and you play for your [GAA] team. Sometimes we do want them to play football, but tradition, values and beliefs come in the way and I think as a society in Ireland, that's our stumbling block”* [NLC8].

Despite this, all participants commented on the benefits of playing other sports throughout childhood and into early adolescence, and no participants expressed a belief that children should focus solely on one sport throughout these formative years. Specifically, twenty-six participants cited the biopsychosocial benefits of playing multiple sports as an important factor in childhood development. In addition, twenty-five were strong advocates of the need for children to sample other sports throughout childhood before they reach their teenage years:

“*I think at seven, eight, nine, ten, eleven [years old] and before they go into the pathway at 13 or 14 for the League of Ireland, and it's the same with the GAA; they've gone 13 as well, I think up to eleven or twelve you should be encouraging it [playing other sports]”* [SC8].

Eleven participants also commented on how they felt that players should be encouraged to recreationally participate in other sports on their non-football days outside of the academy:

“*I do say to them, especially when we're in the off-season, pick up golf, pick up tennis, basketball with your friends, I think all of that is important to do and we do encourage them to do that as long as it doesn't conflict with what we do. We do encourage them. It is important to do other sports”* [NLC6].

### Biological Maturation

Participants were concerned that a selection bias existed throughout the NL in favor of early maturing players. The vast majority of parents and schoolboy coaches explained how they felt that player identification and release/retention was based upon physical attributes and the early maturing players were being preferentially selected into and retained within NL academies:

“*There are some lads and you just look at them and think ‘how is he 14?'. He gets picked because of that. Physically, he may be strong, but technically he may not be that strong. All these guys get lumped into one bucket based on age and the consequence of that is that what coaches look for in that bracket is physicality rather than technical ability”* [P6].

Such a selection bias in favor of early maturing athletes is not uncommon and has been observed within football academies outside of Ireland (i.e., Hill et al., 2020). Nine participants felt that later maturing players were being overlooked in selection for NL academies and were subsequently “falling through the gaps.” Additionally, four participants believed that NL players should be categorized by physical size rather than chronological age. Moreover, ten participants believed that later maturing players were being hindered by the misalignment of age structures within the NL. The age structures of the NL currently run bi-annually from under 15 onwards (i.e., under 15, 17, 19) instead of annually (i.e., under 15–16–17). Participants believed this to be particularly disadvantageous to the later maturing players within the academy pathway, as NLC9 explained:

“*Probably one of the biggest issues that you find, especially going from 15 to 17, is that late developers need to be catered for. That's something I'm seeing at the moment in our under 15s; we have a mixture of sizes and stuff even at this age, and they're all born in the same year. You could have an exceptional under 15, but physically he might be a late developer and he steps up to under 17 level and it's a huge challenge physically and there is a year gap there as well, so not only is he dealing with the physical gap of his own age, but also the physical gap of players the year older.”*

### Systematic Barriers to Player Development

The majority of NL coaches, schoolboy coaches and FAI coaches were all critical of the lack of facilities in the NL, citing it as the biggest organizational barrier to player development in Ireland. NL coaches were particularly critical of the lack of training and match facilities, and expressed the need for intervention across the country:

“*We massively struggle with facilities. I don't know if you've been to [Names location where matches are played], but that needs to be updated to kick players on. I think that would be the case in a lot of clubs around the country. Then Gyms, proper AstroTurf pitches, better quality surfaces to play on. For me, that's a big thing”* [NLC5].

Such concerns were reiterated among FAI coaches:

“*When you look at the National League clubs…I can tell you we've got schoolboy clubs in this county that have their own clubhouse, they've got two all-weather pitches, and they cater for an academy on a Saturday morning of about 400 kids. Then we've got two National League clubs… they don't have a training ground that they own, they don't have a pitch that they own”* [FAIC2].

Schoolboy coaches also criticized the standard and accessibility of facilities in the NL. Schoolboy coaches believed that the standard of facilities in the NL are no better than schoolboy clubs and commented on how many NL clubs were renting training facilities from third parties:

“*You go to [National League Club] and it's like going back in the dark ages, they don't have their own facilities and they train in a public park, it's not even a step up. If you look across the whole of the National League, you could probably look at two or three clubs that have really good facilities. Like [National League Club] don't have any training facilities, they go around and use AstroTurf pitches all around. Really, from a set-up perspective, there is nothing there to entice children to say, ‘this is where I want to be' (…) facilities-wise, it's extremely poor”* [SC4].

Ninety percent of NL clubs share facilities with another club/external source, and when taken in comparison to nations with similar demographics, access to facilities in Ireland is substantially lower (UEFA, 2020). For example, each professional Danish club has nearly double the amount of full-sized grass pitches and has nearly three times the number of artificial pitches than each Irish NL club (UEFA, 2020). Additionally, all NL coaches cited the lack of a full-time football industry in Ireland as a major systematic flaw in the player development system. NL coaches expressed frustrations over the lack of full-time coaching positions within NL academies and how they were expected to manage the development of the highest potential young footballers in Ireland whilst also trying to balance full-time jobs elsewhere:

“*You will find most of the coaches, even though they're highly qualified, they're part-time! Like I work, I finished work today at 2 pm. I've been in since 6 am and I finished at 2, and the nights I train I come home and have something to eat and then I'm on the road again at half 5 and I probably don't get home until 10 that night”* [NLC3].

“*People are always saying we should have academies like in England, like Liverpool's academy. Well, there's no full-time staff, so who coaches these kids all day long? The fact [is] that we don't even have an industry”* [NLC6].

On average, each European premier division club employs 5–7 full-time academy coaches who are exclusively dedicated to youth development (UEFA, 2020). Across all clubs (*n* = 25) and all age cohorts (under 14–19) in the underage NL, there are a total of 6 full-time staff, none of whom are exclusively dedicated to coaching (League of Ireland and FAI Academy Development Manager, personal communication, 2021). In this regard, Ireland is ranked within the lowest threshold in Europe for full-time staff dedicated to organization/logistics, medicine, education, coaching, and scouting/analysis (UEFA, 2020).

In line with this, ten participants were also critical of the lack of financial investment in Irish football. To bridge the gap to other European countries at a senior international level, and to ultimately develop a larger number of high-quality players, participants expressed the need for large scale financial investment in Irish football, as exemplified by FAIC3:

“I think if we look at the National League clubs, even the grassroots clubs, obviously the FAI programmes; the more you put in, the more you get out. And the reality of it is if you look at any of our programmes and you question ‘what do we want to get out of this?', if someone says, ‘we want f^**^cking our senior national team to be qualifying for major European and world finals off the crop of players we have now', you need to put more money in! That's the reality of it. For what the FAI is putting in, and listen, I'm FAI through and through I'll support them until the day I die, but the place is obviously in the sh^*^t at the moment, so what we're putting in is not getting us to world cup finals in 15 years' time, not a chance! Unless every other country stops doing what they're doing, we ain't getting nowhere!”

### Lack of Appropriate Challenge

Participants expressed concerns that the NL were failing to provide appropriate levels of challenge for young players. The importance of providing appropriate levels of challenge in talent development has been emphasized in literature elsewhere (i.e., Collins and MacNamara, 2012; Collins et al., 2016). Specifically, in this Irish football context, fifteen participants from across all stakeholder groups expressed a view that there were large variations between the standard of teams within the NL, characterized by one-sided score-lines and uncompetitive matches. As a consequence, it was believed that players from the stronger teams in the NL were not being challenged appropriately, and the players from teams of lesser quality were being exposed to an environment that was not suited to their needs at that particular stage of their development. Typifying this, SC4 commented:

“*I think if we look at the National League, it's probably not challenging for about three or four clubs who are operating at a totally different level from a National League perspective. They don't get challenged on a week-to-week basis. Some of the children, if they stayed in their local leagues, would have more of a challenge than when they go and play some of the other clubs and win 5, 6, 7, 8–0; that's not a challenge and neither party is learning in that regard.”*

NL coaches also expressed concerns that players were not being challenged frequently enough due to a lack of coaching contact hours and matches. A player aged 12–16 in a category 1 UK football academy gets 12–16 coaching contact hours a week (Read et al., 2016). The consensus among coaches in this study was that players aged 13–15 in a NL academy get 3–6 h per week. This lack of contact hours was considered particularly disadvantageous to player development in Ireland, as NLC6 explained:

“*I think we don't do enough. The contact hours are nowhere near enough. It's crazy to think what everyone else is doing in Europe and players are developing and we're not developing players at the moment in my opinion. I think we don't have enough contact.”*

### An Emphasis on Short-Term Success

Seventeen participants expressed concerns that an emphasis was placed on short-term success over long-term development, opposing a key principle of talent development outlined by Martindale et al. (2007). The majority of parents believed that there was an over-emphasis on winning matches throughout the NL which took priority over player development, as P6 reiterated:

“*You'd like it all not to be so results orientated. You'd love there to be metrics to judge teams based on how they develop players and not how many games that they win. The table is still a table based on how many points a team gets, there's no other table to show other metrics that are important. In our league, it's all about goals, winning and points, but that means then that all the actions that we do are trying to reach them goals.”*

This feeling was reciprocated by several NL coaches:

“*There is an emphasis on winning and some teams and some coaches will win at all costs (…) There's always pressure to win matches. I think in different clubs there's different ethos and value put on winning matches, and sometimes in the National League, it's winning ahead of development”* [NLC4].

## Implications For Practice

Using an extensive sample of stakeholders engaged within Irish underage football, the results of our analyses highlight the lack of horizontal and vertical coherence across the Irish football pathway. This incoherent player pathway and the disjointed relationships between stakeholders were suggested as a significant barrier to long-term player development. Stakeholders appeared to understand talent development principles, but this has not led to the consistent implementation of these principles in the development of young Irish players.

Vertical coherence in Irish football should lead to a symbiotic working relationship between the SFAI, NL clubs, and the FAI. Previous research suggests that football organizations should place value in creating vertically integrated processes of talent development (Webb et al., 2016; Taylor and Collins, 2021). However, vertical integration throughout the pathway in Ireland does not appear to exist. All stakeholders cited how a fractious relationship existed between schoolboy football clubs and NL academies, characterized by conflicts over player transfers, financial disputes and dissonance over the control of player development. This lack of vertical integration was perceived to be intertwined with the poor historical relationship between the FAI and SFAI. The disconnect between the SFAI (an affiliate of the FAI) and the FAI regarding what is best for player development appears a major obstacle for player development in Ireland. Having NGB affiliates self-governing and operating independently from the NGB is not best practice in player development (Webb et al., 2016), particularly when individual leagues within the affiliate operate independently from each other. This lack of vertical coherence throughout the pathway is considered to be an obstacle in long-term player development (Webb et al., 2016; Taylor and Collins, 2021). Indeed, it has been stated that effective talent development environments are distinguished by the existence of a strong organizational structure throughout the pathway (Henriksen et al., 2010a,b). The evident lack of integration between schoolboy football clubs and NL academies was exemplified by the lack of shared understanding regarding coaching and the purpose of the NL academies, and ultimately, the purpose of the player pathway. In theory, a shared understanding of player development in Ireland would entail schoolboy clubs working closely with NL clubs to facilitate the smooth transition of players from the schoolboy level into the academy environment. Whilst this shared understanding and vertically coherent player development system ceases to exist, the player development pathway within Irish football is unlikely to be optimized (Mills et al., 2014a).

A lack of horizontal coherence was also apparent at the NL level, most notably in the relationships between coaches and parents. The existence of horizontal coherence in an academy context should see academy coaches and parents working with each other and with the player in an agreed fashion to optimize the player development experience (Taylor and Collins, 2021). However, the academy pathway was characterized by poor communication and a lack of role clarity between coaches and parents. Parents expressed concerns over the lack of regular and comprehensive communication from coaches, which is a common concern within football academies elsewhere (Harwood et al., 2010; Clarke and Harwood, 2014). On the other hand, coaches explained how parents can often interfere with coaching practices, most notably by providing inappropriate and contradictory coaching advice (in line with Mills et al., 2012). Such findings indicate that parents often appear unaware of their role (Côté, 1999) and suggest the need for academies to implement regular and comprehensive parent education workshops (Clarke and Harwood, 2014; Newport et al., 2020). Academies should work with parents to build more positive working relationships tailored toward optimizing their influential role as “football parents” (cf. Mills et al., 2012).

Multiple stakeholders raised concerns over the youth to senior transition within the player pathway. Participants explained how academy and senior team operations are not aligned and how the transition from academy to first-team football is too great for many players. Significantly, the youth to senior athlete transition has traditionally been noted as the most challenging transition in an athlete's career (Stambulova et al., 2009) and dichotomy between the two stages of the pathway is likely to have detrimental repercussions for successful player development (Mills et al., 2014b). In a NL context, it appears that youth and senior team operations and transitions are not well-integrated.

Despite the lack of stakeholder coherence across and within the pathway, talent development principles between the stakeholders appear well-understood. For instance, participants highlighted the need to provide an appropriate and individualized challenge for players throughout the pathway (Collins and MacNamara, 2012) and noted the need to emphasize long-term development over short-term success (Martindale et al., 2007). Despite this, as is the case in many talent development pathways (Pankhurst et al., 2013), the implementation of these talent development principles in an applied context does not appear to exist. In this respect, participants were critical of the academy system and explained how players were not being provided with appropriate or individualized levels of challenge. Stakeholders perceived there to be large variations in the quality of academy teams, characterized by uncompetitive matches and uneven score-lines. Moreover, there was a common perception among stakeholders that a selection bias existed in favor of early maturing players and that this may have a detrimental impact on long-term player development (cf. Cumming et al., 2018).

In line with the key principles of effective talent development environments (Martindale et al., 2007), participants were critical of the emphasis that was placed upon winning matches at the academy level. The use of short-term success as a marker of effectiveness within the NL appears short-sighted, especially given that the system is suggested to specifically focus on producing Irish internationals and senior first-team club players. Fundamentally, football academies exist to produce individual players, not successful academy teams (Mills et al., 2012). Without a change in emphasis on winning academy football matches, problems will continue to exist in player selection, coaching, and the subsequent experiences of the academy players (Martindale et al., 2007).

Participants described how they believed an early engagement in football to be optimal for player development. They also emphasized the importance of devoting the majority of childhood activity to football to maximize development before entering an academy at age 13. The importance of experiencing large amounts of football-specific learning activities during childhood has been emphasized in the literature elsewhere (Ford et al., 2009; Ford and Williams, 2012; Sieghartsleitner et al., 2018; Zibung and Conzelmann, 2013). However, participants believed that this should not be at the expense of participation in other childhood sporting activities particularly up to 12 or 13 years of age. Crucially, participants acknowledged the unique context in which the Irish player pathway exists and the cultural dominance of the GAA. Indeed, culture has an over-arching emphasis on the development of talent (Martindale et al., 2007; Henriksen et al., 2010a). In this respect, implementing a player development system similar to those seen in other football nations (i.e., Elite Player Performance Plan, 2011) may not be viable nor appropriate in an Irish-specific context as it may conflict with cultural and societal norms.

The question for Irish football is whether Ireland can consistently produce players capable of competing at the highest level of international football when young players develop in a cultural climate where football exposure time is limited due to competitive participation in multiple sports until mid-late teens. The benefits of this approach in football player development remain unclear (cf. Sweeney et al., 2021). Between 2011 and 2020, out of all the 200 FIFA member associations, Ireland had the most players aged under 18 transferred to professional clubs abroad, most consistently to UK-based clubs (FIFA, 2021a). This is likely a reflection of the standard of the domestic league in Ireland during that time period (ranked between 31st and 43rd out of a maximum of 55 European leagues; UEFA, 2021). It was in these UK-based academies where the highest potential Irish players continued their development after leaving Ireland. However, once the UK formally left the EU during Brexit, the exemption that allows European clubs to sign European players aged under 18 no longer applied to UK-based clubs, making Irish clubs responsible for developing players up to 18 years of age. With a lack of vertical and horizontal stakeholder coherence, coinciding with a lack of full-time staff, facilities, financial investment, and football exposure time, Irish football is arguably faced with its biggest ever challenge to develop players capable of competing against its international counterparts. Ireland's senior international men's team is currently ranked 51st out of 210 nations, compared to a ranking of 28th only 10 years ago (FIFA, 2021b). From the start of the 2021/22 season, there were 22 Irish players registered with an English Premier League first team (The Irish Times, 2021), with no capped Irish players registered with a first team in any other top-five ranked European domestic football league (La Liga, Serie A, Ligue 1, Bundesliga; UEFA, 2021).

### Limitations

Given that the focus of this research was on the Irish football player pathway, results and their implications are specifically limited to an Irish football context. In this respect, findings may not apply to non-Irish contexts or alternative sporting pathways. It is up to the reader to consider the transferability of these findings to their specific sporting and cultural context. However, given the recent restructure of the Irish football player pathway and the introduction of the newly formed underage NL, it was crucial from both an organizational and practical perspective to explore stakeholder perceptions of the player development experience. This was the first research of its kind to comprehensively analyse the Irish football player pathway from the perspectives of the key stakeholders. Given the extensive (*n* = 31) and contemporary participant sample of this research, along with the nature of data collection (one-to-one interviews to facilitate an in-depth and individualized investigation), we believe these findings to be valid and reliable. In this regard, results can be utilized to guide decisions on future player and coach development policies within the FAI. An additional limitation of this research was that it focused on the Irish player pathway for male players only. Although some participants who were interviewed in this research study were female, it is clear that the research focus lacks gender diversity.

## Future Directions

We have examined the experiences and perceptions of the key stakeholders invested in the player pathway. As a next step, it is important to consider the experiences and viewpoints of the young Irish players engaged in the pathway themselves. To date, young players have been absent from the discussion. To optimize the pathway and player development policies, discussions must take place with the players engaged within the academy pathway, across multiple Irish football academies and age cohorts. It would be of particular value to identify the players' perceptions of the quality of their talent development environment and optimal developmental practices. It is also important to examine the players' perceptions of their relationships with the different stakeholders involved in the development process. The additional benefit of this approach is clear, and we will seek to examine these perceptions in our upcoming research agenda.

## Data Availability Statement

The raw data supporting the conclusions of this article will be made available upon request to the corresponding author.

## Ethics Statement

The studies involving human participants were reviewed and approved by Dublin City University Research Ethics Committee. The patients/participants provided their written informed consent to participate in this study.

## Author Contributions

LS, DH, and ÁM contributed to the conception and design of the study. LS performed the data collection and data analysis, with ÁM reviewing the analysis. LS wrote the initial manuscript, with DH and ÁM contributing to manuscript revisions. All authors read and approved the submitted manuscript.

## Funding

The research conducted in this publication was funded by the Irish Research Council under award number GOIPG/2021/277.

## Conflict of Interest

DH was employed by company Football Association of Ireland. The remaining authors declare that the research was conducted in the absence of any commercial or financial relationships that could be construed as a potential conflict of interest.

## Publisher's Note

All claims expressed in this article are solely those of the authors and do not necessarily represent those of their affiliated organizations, or those of the publisher, the editors and the reviewers. Any product that may be evaluated in this article, or claim that may be made by its manufacturer, is not guaranteed or endorsed by the publisher.

## Footnotes

^1^DDSL: The Dublin District Schoolboy League is the largest of the thirty schoolboy football leagues in Ireland.
